# Quantitating Fluorescence Intensity from Fluorophore: The Definition of MESF Assignment

**DOI:** 10.6028/jres.107.009

**Published:** 2002-02-01

**Authors:** Abe Schwartz, Lili Wang, Edward Early, Adolfas Gaigalas, Yu-zhong Zhang, Gerald E. Marti, Robert F. Vogt

**Affiliations:** Center for Quantitative Cytometry, PO Box 194344, San Juan, Puerto Rico 00919; National Institute of Standards and Technology, Gaithersburg, MD 20899-8312; Molecular Probes, Inc., PO Box 22010, Eugene, OR 97402-0469; Center for Biologics Evaluation and Research, U.S. Food and Drug Administration, NIH Building 29B, Room 2NN08, Bethesda, MD 20892; Division of Laboratory Sciences, CDC, Mailstop F19, 4770 Buford Highway, Atlanta, GA 30341

**Keywords:** flow cytometer, fluorescein, fluorescence, MESF, microbeads, quantitation, SRM 1932

## Abstract

The quantitation of fluorescence radiance may at first suggest the need to obtain the number of fluorophore that are responsible for the measured fluorescence radiance. This goal is beset by many difficulties since the fluorescence radiance depends on three parameters 1) the probability of absorbing a photon (molar extinction), 2) the number of fluorophores, and 3) the probability of radiative decay of the excited state (quantum yield). If we use the same fluorophore in the reference solution and the analyte then, to a good approximation, the molar extinction drops out from the comparison of fluorescence radiance and we are left with the comparison of fluorescence yield which is defined as the product of fluorophore concentration and the molecular quantum yield. The equality of fluorescence yields from two solutions leads to the notion of equivalent number of fluorophores in the two solutions that is the basis for assignment of MESF (Molecules of Equivalent Soluble Fluorophore) values. We discuss how MESF values are assigned to labeled microbeads and by extension to labeled antibodies, and how these assignments can lead to the estimate of the number of bound antibodies in flow cytometer measurements.

## 1. Introduction

The number of fluorescence based assays has grown rapidly in the last 20 years, especially in the fields of biological research and clinical diagnosis [1]. The growth has been driven by the rapid developments in fluorophore conjugation chemistry [2] and by the high inherent sensitivity of fluorescence measurement (down to a few hundred fluorophores or less) [3, 4]. With the proper use of standards the fluorescence intensity can be quantitated yielding further utility to fluorescence based assays [5–7].

Soluble analytes can be quantitated using reference materials dissolved in the same media as the analyte. In such cases the molar extinction and quantum yield of the fluorophore is the same for the analyte and the reference material and the fluorescence radiance from both is proportional to the relative number of fluorophores. In the case where one set of fluorophores is in solution and the other set of fluorophores is immobilized on a microbead, the quantum yields may be different for the two fluorophores and the equality of fluorescence radiance does not imply equal numbers of fluorophores. In what follows we discuss the notion of MESF (Molecules of Equivalent Soluble Fluorophore) as a method for quantitating fluorescence radiance in the case where quantum yields differ between the analyte and the reference solution.

The discussion below is based on the concept of fluorescence yield which is defined as the product of the fluorophore concentration and the quantum yield of the fluorophore. The quantum yield is defined as the ratio of the number of photons emitted by a fluorophore to the number of photons absorbed by the fluorophore. In the same spirit the fluorescence yield of a solution is a measure of the number of photons emitted per unit volume if every fluorophore absorbs a photon. For fixed solution conditions the fluorescence yield is a well-defined property of the solution. Just as it is valid to compare the quantum yield of two fluorophores, it is also valid to compare the fluorescence yield of any two solutions. Experience has shown that the quantum yield and hence the fluorescence yield is a highly variable property of the fluorophore’s microenvironment. For example, due to the existence of multiple forms of fluorescein, the apparent quantum yield of fluorescein changes from 0.93 to 0.37 when the buffer pH goes from 9 to 5 [8,9]. Similarly the fluorescence yield of the solution would change upon a change in pH. Conjugating a fluorophore to a protein [10] or DNA [11] usually changes its quantum yield. Hence it was realized early that trying to quantitate fluorescence intensity in terms of an actual number of fluorophores is a difficult task. The notion of fluorescence yield allows solutions to be compared in a meaningful way and is the basis for the notion of MESF which has been used as a practical method for quantitating fluorescence radiance. In what follows we will discuss the concept of fluorescence yield, how it leads to MESF, the actual assignment of the MESF value, and finally some cautionary remarks on the variation expected in practical assignment of the MESF values.

## 2. Comparison of Fluorescence Yields

**Fig. 1:**
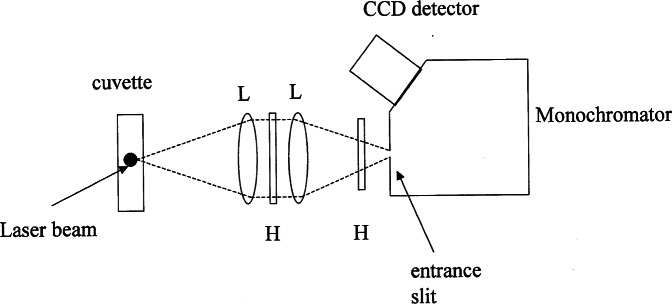
A schematic diagram of the apparatus used to measure the fluorescence intensity from a solution (suspension) in a cuvette or a flow thru cell. The laser beam is coming out of the plane of the figure and is represented by the dot in the cuvette. The pair of lenses (L) focus the illuminated region in the cuvette on the monochromator entrance slit (M=1). The holographic notch filters (H) are tuned to reject the laser radiation wavelength, in this case 488 nm. The entrance slit is imaged and dispersed on the CCD detector.

The fluorescence yields will be compared relative to a standard reference solution of a fluorophore such as fluorescein. The reference solution will serve as a “meter stick” to which all other fluorescence yields will be compared. The fluorescence yield will be obtained from a measurement of fluorescence radiance. In the following we use the term “fluorescence intensity” and fluorescence signal interchangeably, both denote the output of a detection system that measures the radiation from a fluorescing sample. The measurements will be carried out using a conventional fluorimeter consisting of a monochromator with a CCD detector at the exit slit, and a source consisting of a cuvette with a illumination region parallel to the entrance slit of the monochromator. A schematic of the apparatus is shown in Fig. 1. The measurement of the fluorescence radiance will be performed by combining the fluorescence signal from the fluorescing source at wavelength *λ*_0_, *S*_f_(*λ*_0_), and signal from a radiance standard, *S*′(*λ*_0_). The two measured signals will be combined as shown in Eq. (1) where *L′*(*λ*_0_) is the known radiance of the standard source and *L*_f_(*λ*_0_) is the unknown radiance of the fluorescing sample. This combination of the measured signals provides a correction for instrument spectral response. The measured fluorescence spectral radiance is given in terms of measured signals by (Eq. (A13) in the Appendix)

$$L_{f}\left(\lambda_{0}\right)=\frac{S_{f}\left(\lambda_{0}\right)}{S^{\prime }\left(\lambda_{0}\right)}L^{\prime }\left(\lambda_{0}\right).$$ (1)

As shown in the appendix [Eq. (A10)], the spectral radiance *L*_f_(*λ*_0_) can also be given in terms of the properties of solution and fluorophore by

$$L_{f}\left(\lambda_{0}\right)=I_{0}N\Omega \varepsilon \left(\lambda_{x}\right)\phi s\left(\lambda_{0},\lambda_{x}\right)$$ (2)

where *I*_0_ is the incident power (W), *N* is the concentration of fluorophores (1/m^3^), *Ω*(1/m sr) is a factor depending on the geometry of the source and the detector, *ε*(*λ_x_*) is the absorptivity (m^2^), *ϕ* is the quantum yield, *s*(*λ*_0_, *λ_x_*) is the relative spectral emission function (1/nm). The relative emission function, *s*(*λ*_0_, *λ_x_*) will be normalized so that the integral over all wavelengths is 1. In many cases the emission spectrum is independent of the excitation wavelength and can be written as a product of a quantum yield and a normalized relative emission function [see Appendix Eq. (A14)]. Physically, when a molecule is excited, some of the energy goes into changing the state of the electron, and the remainder goes into nuclear vibrations. The nuclear vibrations usually dissipate their excess energy to the surroundings and reach the lowest vibrational state in less than a ps. Thus the emission always occurs from the same equilibrated excited state even if the initial excited state at the instant of photon absorption was different. Molecules in the equilibrated excited states have many relaxation pathways and changes in the relative importance of these pathways may perturb the emission spectrum. We will assume that the emission spectrum is independent of the other non radiative relaxation pathways. The quantum yield is a measure of the relative probability that the excitation will relax via a radiative decay (fluorescence) versus the non radiative pathways. In what follows we will compare the fluorescence radiance, *L*_f_(*λ*_0_) of two sources that may have different excitation probabilities, different relative emission functions *s*(*λ*), and different quantum yields, *ϕ*.

The total fluorescence radiance is obtained by summing over all wavelengths resulting in an expression

$$L_{f}=\int \frac{S_{f}\left(\lambda_{0}\right)}{S^{\prime }\left(\lambda_{0}\right)}L^{\prime }\left(\lambda_{0}\right)d\lambda_{0}=\Omega N\phi \varepsilon \left(\lambda_{x}\right)I_{0}.$$ (3)

In writing Eq. (3), we have used the fact that the sum of *s*(*λ*_0_, *λ_x_*) over all values of *λ*_0_, gives 1 according to the definition of *s*(*λ*_0_, *λ_x_*) in the Appendix. Suppose that we are free to adjust the concentration of fluorophore in one of the solutions and we adjust the concentration so that the two fluorescing sources give the same total fluorescence radiance. Then according to Eq. (3) we can write

$$\begin{array}{c} L_{s1}=L_{s2}\\ I_{0}N_{s1}\Omega \varepsilon_{s1}\left(\lambda_{x}\right)\phi_{s1}=I_{0}N_{s2}\Omega \varepsilon_{s2}\left(\lambda_{x}\right)\phi_{s2} \end{array}$$ (4)

where *L*_s1_ and *L*_s2_ are the total fluorescence radiance [Eq. (3)] measured for two fluorophore solutions or suspensions named s1 and s2, respectively. We say that the response of *N*_s1_ molecules of fluorophore s1 per m^3^ is equivalent to the response of *N*_s2_ molecules of fluorophore s2 per m^3^. Assuming that the geometric factor, *Ω*, and the illumination, *I*_0_, are the same for both the solution measurements, the equality of total fluorescence radiance implies the following relation

$$N_{s1}\varepsilon_{s1}\left(\lambda_{x}\right)\phi_{s1}=N_{s2}\varepsilon_{s2}\left(\lambda_{x}\right)\phi_{s2}.$$ (4a)

**Fig. 2:**
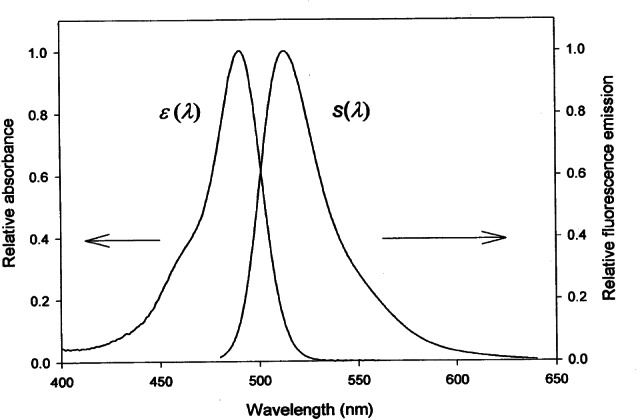
The measured relative absorption and relative fluorescence emission of fluorescein in a pH 9 borate buffer. The emission spectrum was taken with the apparatus shown in Fig. 1, and the excitation spectrum was taken with a SLM 8000 spectrofluorimeter. The relative emission function *S*(*λ*) was obtained by dividing the detector response by the response at 515 nm. The integral of the function *S*(*λ*) shown in the figure is not normalized to 1.

Equation (4a) shows the well known fact that the fluorescence radiance is determined by the product of three independent variables: the number of fluorophores, the probability of absorbing a photon, and the probability of emitting a photon. Thus if the above measurement is repeated with a different illumination wavelength, the fluorescence radiances will not be equal since the molar absorptivity will be different. Figure 2 shows the wavelength dependence of the relative absorption of fluorescein in a pH 9 solution. The absorption is a strong function of wavelength and thus the resulting integrated fluorescence radiance will depend on the wavelength of illumination. In order to eliminate the dependence on excitation wavelength, the fluorescence radiance measurement needs to be normalized by molecular absorptivity at the illumination wavelength. Such a normalization leads to a quantity known as fluorescence yield which is defined as the product of fluorophore concentration and the molecular quantum yield. If a series of measurements of fluorescence yield are performed for different concentrations of one of the fluorophores then equality of the measured fluorescence yields implies the relationship

$$N_{s1}\phi_{s1}=N_{s2}\phi_{s2}.$$ (4b)

The equality of the measured fluorescence yields would be instrument independent. All instruments that measure the ratio of fluorescence radiance and molecular absorptivity would give the same response for solutions having identical fluorescence yields [Eq. (4b)]. The equality would be true even if the absorption and emission spectra are very different for the two fluorophores. A practical procedure for converting a comparison of fluorescence radiance into a comparison of fluorescence yields is to work with the same fluorophore in the standard solution and in the analyte so that the molecular absorptivities are reasonably matched and cancel out from the comparison of fluorescence radiances. The equality of fluorescence radiance is then equivalent to equality of fluorescence yield, [Eq. (4b)]. An alternative approach would be to design fluorophore whose molecular absorptivity did not depend on wavelength or solvent, e.g., some nanocrystals.

Suppose we compare the fluorescence yield of two solutions or suspensions. If the fluorescence yield is the same for the two solutions and if the concentration of fluorophore is known for the two solutions we say that the fluorescence yield of *N*_s1_ molecules of fluorophore s1 per m^3^ is equivalent to the fluorescence yield of *N*_s2_ molecules of fluorophore s2 per m^3^. This equivalency is based on the equality of solution properties (fluorescence yield) and does not depend on the instrument. The equivalency gives a measure of the number of fluorophores in one solution (analyte) relative to a number of fluorophores in another solution (reference); this is the basis for the assignment of MESF value.

## 3. Assignment of MESF Value to Microbeads

In the following, we will describe the procedure for assigning a MESF value to a microbead with immobilized fluorophores. We will equate the fluorescence yield of the microbead suspension to the reference solution. In what follows we assume that the reference solution and the microbeads have the same fluorophore and that the molecular absorptivity is the same for the two. The solution and suspension are prepared with known number concentration of soluble fluorophores, *N*_sol_, and number concentration of microbeads, *N*_b_. Since we assume that fluorescein in both environments has the same molecular absorptivity, the equality of fluorescence radiance is equivalent to equality of fluorescence yields.

$$N_{\mathrm{sol}}\phi_{\mathrm{sol}}=N_{b}\phi_{b}.$$ (5a)

Using the reference solution as the unit of fluorescence yield we can assign a value of MESF to the microbead as

$$MESF=\left(\frac{N_{A}}{1000}\right)\frac{N_{\mathrm{sol}}}{N_{b}}.$$ (5b)

**Fig. 3:**
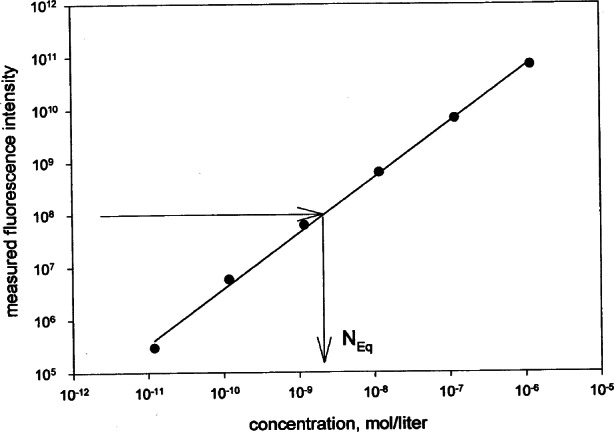
A graphical display of the equivalence of fluorescence yields of a microbead suspension and reference fluorophore solution. The straight line is a fit to the six points representing the measured fluorescence radiance in six dilutions of the reference solution. The horizontal arrow points to the measured fluorescence yield of the microbead suspension, and the vertical arrow points to the equivalent concentration of soluble fluorophores.

The factor *N*_A_/1000 converts number concentration into a molar concentration, *N*_A_ is the Avogadro constant, and 1000 is the number of cm^3^ in a liter (we use cm^3^ to conform to laboratory practice). An example of the above procedure is shown in Fig. 3 where the two arrows show a graphical display of the equivalence of fluorescence yield from a microbead suspension (horizontal arrow) and the concentration of the reference solution, *N*_sol_ = *N*_eq_ (mol/L), which gives an equal fluorescence yield. The fluorescence yield of the solution is obtained from a straight line fitted to measured fluorescence yield of six serial dilutions of the reference solution. The number concentration of microbeads was measured using a Coulter particle counter and gives a number *N*_b_ (1/cm^3^). The values *N*_eq_, and *N*_b_ are inserted into Eq. (5b) to yield the value of MESF for the microbead. The value of MESF gives the number of soluble fluorophores which gives the same fluorescence yield as the fluorophores immobilized on a single microbead. The MESF value accounts for the change in quantum yield between fluorophores in solution and immobilized on the microbead.

The procedure for assigning MESF values to microbeads requires accurate measurement of the particle concentration and emission spectra. The particle concentration can be obtained using a Coulter particle counter. The emission spectra are difficult to measure for the microbeads with low number of immobilized particles. Scattering can be eliminated by using two holographic notch filters in the apparatus (Fig. 1). The background contribution from the buffer solution has to be subtracted with care (a buffer solution has several constituents in order to maintain a constant pH). The emitted (fluorescence) photons can also scatter from the microbeads in solution and thus either increase or decrease the observed fluorescence intensity. The biases due to fluorescence scattering have to be investigated. Performing the measurements on a set of reference solutions and a set of microbeads with different number of immobilized fluorophore, we obtain a set of microbeads with different values of MESF. These microbeads can be used to calibrate the response of a flow cytometer.

## 4. Application of MESF Units

The assignment of MESF values to the microbeads is independent of the spectral properties of the fluorophores in reference solution and on the microbead. This was ensured by integrating over the entire corrected emission spectrum [see Appendix Eqs. (16A) and (17A)]. However, when microbeads are applied to calibrating a cytometer, the measuring instrument may sample over a limited range of emission wavelengths. Furthermore the instrument response will usually not be corrected for spectral response. The signal, *S*_f_(*λ*_0_), of an instrument (photomultiplier tube or CCD) can be modeled using Eq. (A12) in the Appendix:

$$S_{f}\left(\lambda_{0}\right)=L_{f}\left(\lambda_{0}\right)\;\;\Gamma \;R\left(\lambda_{0}\right)\;\;\Delta \lambda_{m}.$$ (6)

*L*_f_(*λ*_0_) is the spectral radiance of the source, and Δ*λ*_m_ is the bandwidth of the emission, Γ is the throughput of the detection system and the function *R*(*λ*) describes the responsivity of the detection system. To be specific, we assume that the instrument is a flow cytometer so that *N* represents the number of fluorophores on the particle passing the detection region. In order to quantify the response of a biological cell we find a microbead such that the fluorescence signals of the cell and the microbead are equal. The equality of the two signals is modeled by the following equation derived from Eqs. (2) and (6):

$$N_{C}\varepsilon_{\mathrm{ex},C}\phi_{C}\int R\left(\lambda \right)s_{C}\left(\lambda \right)d\lambda =N_{b}\varepsilon_{\mathrm{ex},b}\phi_{b}\int R\left(\lambda \right)s_{b}\left(\lambda \right)d\lambda .$$ (7)

The function *s*(*λ*) is the relative spectral emission, and the subscripts *C* and *b* stand for biological cell and calibrated microbead, respectively. The above equation models the situation where the cell and the microbead give the same response on the instrument. The equality of the two fluorescence signals is used to assign the same MESF value to the cell as the microbead. Equation (7) points to the fact that the assigned MESF value depends on the properties of the instrument’s detection system, *R*. This could lead to problems since the assigned values of MESF would be instrument dependent. The instrument factor drops out of Eq. (7) if the fluorophores on the analyte and microbead have the same spectral functions *s*(*λ*). Furthermore if the fluorophores on the analyte and microbead have the same extinction coefficient, Eq. (7) reduces to a equality of fluorescence yields (products of fluorophore number and quantum yield).

$$N_{C}\phi_{C}=N_{b}\phi_{b}.$$ (8)

The biological cell has an assigned value of MESF which is the same as that of the microbead because both have the same fluorescence yield. However, the actual number of fluorophores may not be the same on the two objects.

In practice, the buffer (for biological cells and microbeads) that is used in a cytometer measurement is different from the buffer (for microbeads and the Standard Reference Material, SRM) that is used in the assignment of MESF values to the microbeads Eq. (5a). In order that the assignment of MESF value to the microbead be independent of the buffer used, the quantum yields in Eq. (5a) have to change proportionately in different buffers. This is an important assumption underlying the utility of MESF concept. However if the microbead MESF assignment is independent of the buffer, then Eq. (8) provides a valid transfer of the microbead MESF value to the biological cell.

In summary, we restate that the MESF value is assigned to the biological cell using the criteria that the fluorescence yields are the same for the biological cell and the microbead with which it is compared. The fluorescence yield is equivalent to fluorescence intensity only if molecular absorptivities are the same (the absorption spectra match or have been used to normalize the fluorescence intensity). The value of MESF assigned to the biological cell will be independent of the instrument if the microbead and the cell are measured on the same instrument with the same settings, and the emission spectra match. In a subsequent paper we use Eq. (7) to estimate the variation in the assigned MESF values if the microbeads and biological cells have slightly different absorption and emission properties, and if the instruments have different filters.

## 5. Utility of MESF

The fluorescence from biological cells originates from fluorophores conjugated to antibodies which are bound to receptors (antigen) present on the walls of the biological cell. Using the same procedure that was described for microbeads, it is possible to assign MESF values to antibodies with conjugated fluorophores (labeled antibodies). It is a good assumption that the value of MESF assigned to the labeled antibodies does not change appreciably when the antibodies are bound to cells. Thus the value of MESF assigned to the entire cell in a flow cytometer can be equated to the product of MESF of the individual labeled antibodies and the number of antibodies on the cell. Dividing the value of the MESF assigned to the cell by the MESF of a single antibody would yield the actual number of antibodies on the biological cell which is the number of interest to the biologist. It is important to note that the number of antibodies bound to the surface of the biological cell is not necessarily equivalent to the number of receptor (antigen) molecules expressed on the biological cell (antigen density). The relationship between the amount of bound antibody and the amount of receptors (antigen) on the biological cell is complicated by many factors, and therefore careful experiments are required to reach an accurate assessment of that relationship.

## 6. Conclusion

We developed a conceptual framework for interpreting fluorescence radiance measurements and introduced the notion of fluorescence yield which is the measure fluorescence radiance divided by the molecular absorptivity. We then showed that the equivalence of fluorescence yields of two solutions leads to the notion of equivalent number of fluorophores in the two solutions. This is the basis for assigning a MESF value for one solution or suspension in terms of a reference solution. The concepts were applied to quantitating flow cytometer measurements by the following procedure. First, MESF values are assigned to the microbeads with immobilized fluorophores using a standard reference solution. Second, a cytometer is used to compare the fluorescence intensity of microbeads and biological cells with bound labeled antibodies. Equality of fluorescence intensity is then used to determine MESF values of the biological cells. MESF is a stoichiometric unit for the fluorescence intensity of labeled particles or cells expressed as the number of fluorophore molecules in solution required to produce the same fluorescence yield as that measured on the labeled particles or cells. For example, if a cell labeled with fluorescein emits as much fluorescence as 5000 molecules of fluorescein in solution, it is assigned a value of 5000 MESF. However, this does not mean that the cell is labeled with 5000 fluorescein molecules, but rather with enough molecules of fluorescein to have the same level of emission as 5000 molecules of fluorescein in solution under similar conditions.

The procedure used for cytometers could be modified and extended to quantitating fluorescence intensity measurements in DNA microarray.

## Figures and Tables

**Fig. 4:**
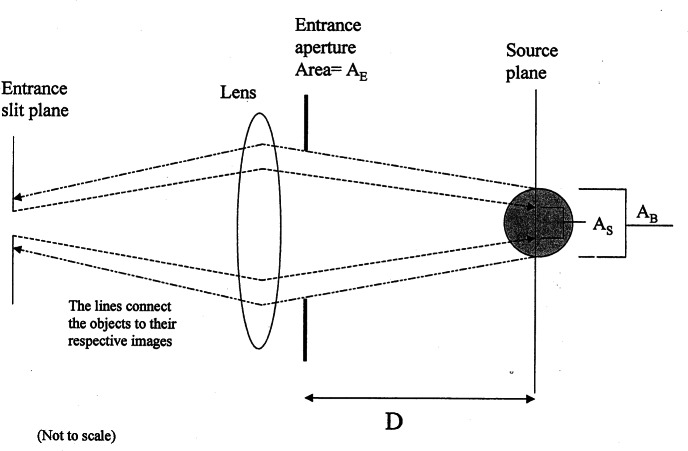
A schematic diagram of the geometry that is used to convert a volume fluorescence source into a equivalent surface source. *A*_B_ is the cross sectional area of the illuminating laser beam which is coming out of the plane of the figure. The source plane intersects the illuminating beam at its mid section. *A*_E_ is the area of the entrance aperture of the collecting optics, and *D* is the distance between the entrance aperture and the source plane. Finally, *A*_S_ is the area of the image of the monochromator entrance slit on the source plane. (*A*_S_ < *A*_B_). The refraction at the cuvette wall is not shown in the figure. A radiance standard would replace the cuvette during calibration. The surface of the radiance standard is placed at the source plane.

## 7. Appendix A. Derivation of the Measurement Equation

The experimental setup, execution, and analysis of measurements involving optical radiation are greatly simplified by using a measurement equation. This equation mathematically describes the measurement, relating the signal from the detector to the optical properties of the incident light, sample, and detection system. The measurement equation for the total fluorescent radiance is derived in this Appendix in a four-step process. In the first three steps equations are derived for the fluorescence radiant flux, the signal from the detection system, and the fluorescent spectral radiance. The last step combines all of the concepts into a measurement model for fluorescence radiance. Much of the derivation is based upon concepts presented by K. D. Mielenz, “Photoluminescence Spectrometry” in Optical Radiation Measurements, Volume 3, Measurement of Photoluminescence, ed., K. D. Mielenz, Academic Press (1982) pp. 2–87.

### 7.1 Fluorescence Radiant Flux

Any measurement of optical radiation involves spectral fluxes over finite spectral bandwidths. In the case of fluorescence, fluxes of both excitation and emission are present. These are denoted by subscripts x and m, respectively. The radiant flux absorbed by a sample, whether it is fluorescent or not, is given by

$$\Phi_{a}\left(\lambda_{x}\right)\Delta \lambda_{x}=\Phi_{i}\left(\lambda_{x}\right)\Delta \lambda_{x}\left(1-10^{-\varepsilon \left(\lambda_{x}\right)NI}\right),$$ (A1)

where *λ*_x_ (nm) and Δ*λ*_x_ (nm) are the wavelength and spectral bandwidth of the excitation radiant flux, respectively, Φ_a_ (W/nm) and Φ_i_ (W/nm) are the absorbed and incident spectral fluxes, respectively, *ε*(cm^2^) is the absorptivity, *N* (1/cm^3^) is the concentration of fluorophore, and *l* (cm) is the path length. Expanding the expression in parentheses and keeping only the first-order term, Eq. (A1) becomes

$$\Phi_{a}\left(\lambda_{x}\right)\Delta \lambda_{x}=2.3Nl\varepsilon \left(\lambda_{x}\right)\Phi_{i}\left(\lambda_{x}\right)\Delta \lambda_{x}.$$ (A2)

The radiant flux from fluorescence is a fraction of the excitation radiant flux, and is given by

$$\Phi_{f}\left(\lambda_{m},\lambda_{x}\right)\Delta \lambda_{m}=y\left(\lambda_{m},\lambda_{x}\right)\Delta \lambda_{m}\Phi_{a}\left(\lambda_{x}\right)\Delta \lambda_{x},$$ (A3)

where *λ*_m_ (nm) and Δ*λ*_m_ (nm) are the wavelength and spectral bandwidth of the emission radiant flux, respectively, Φ_f_ (W/nm) is the fluorescent spectral flux, and *y* (1/nm) is the spectral quantum yield. Note the *y* relates the absorbed radiant flux at wavelength *λ*_x_ to the fluorescent radiant flux at wavelength *λ*_m_. Using the expression for the absorbed radiant flux from Eq. (A2), Eq. (A3) becomes

$$\Phi_{f}\left(\lambda_{m},\lambda_{x}\right)\Delta \lambda_{m}=2.3Nly\left(\lambda_{m},\lambda_{x}\right)\Delta \lambda_{m}\varepsilon \left(\lambda_{x}\right)\Phi_{i}\left(\lambda_{x}\right)\Delta \lambda_{x}.$$ (A4)

### 7.2 Relation Between Detector Signal and Source Spectral Radiance

Figure 4 shows a schematic diagram of an aperture system for a measurement system consisting of a wavelength selector and an optical detector (not shown in Fig. 4), be it a filter and detector, a monochromator and a single detector, or a monochromator and an array detector. The signal at a given wavelength setting of the detection system is given by

$$S\left(\lambda_{0}\right)=\int \Phi_{D}\left(\lambda_{m}\right)R\left(\lambda_{0},\lambda_{m}\right)d\lambda_{m}.$$ (A5)

Here, *λ*_0_ (nm) is the wavelength setting of the detection system, *S* (A) is the signal, Φ_D_ (W/nm) is the spectral flux entering the detector, *R* (A/W) is the responsivity whose dependence on both *λ*_0_ and *λ*_m_ is shown explicitly, and *λ*_m_ (nm) is the wavelength of the emission radiant flux. For any wavelength selector with a narrow spectral bandwidth, such as a monochromator, *R*(*λ*_0_, *λ*_m_) is a sharply peaked function about *λ*_0_, in which case Eq. (A5) becomes

$$S\left(\lambda_{0}\right)=\Phi_{D}\left(\lambda_{0}\right)R\left(\lambda_{0}\right)\Delta \lambda_{m}.$$ (A6)

The spectral flux entering the detector is related to the spectral radiance of the source by

$$\begin{array}{cc} \Phi_{D}\left(\lambda_{0}\right)=L\left(\lambda_{0}\right) & \Gamma =L\left(\lambda_{0}\right) \end{array}\frac{A_{E}A_{S}}{D^{2}},$$ (A7)

where *L* [W/(m^2^ nm sr)] is the spectral radiance, *A*_E_ (m^2^) is the area of the entrance aperture of the collection optics, *A*_S_(m^2^) is the area of the image of the entrance slit in the source plane, *D* (m^2^) is the distance between the entrance aperture and the source plane, and *I* (m^2^ sr) is defined by Eq. (A7). Using Eq. (A7), Eq. (A6) becomes

$$S\left(\lambda_{0}\right)=L\left(\lambda_{0}\right)\Gamma R\left(\lambda_{0}\right)\Delta \lambda_{m}.$$ (A8)

### 7.3 Fluorescent Spectral Radiance

The fluorescence source in a cuvette measurement is a volume source with dimensions set by the illuminating beam. Radiance standards are surface sources with known radiance per unit area of the surface. To compare the fluorescence source to the standard source we need to convert the volume source into an equivalent surface source. The spectral radiance from fluorescence source can be defined in terms of the spectral flux from fluorescence, Eq. (A4), as

$$\begin{array}{c} L_{f}\left(\lambda_{m},\lambda_{x}\right)\Delta \lambda_{m}=\frac{f}{4\pi }\frac{1}{n_{S}A_{B}}\Phi_{f}\left(\lambda_{m},\lambda_{x}\right)\Delta \lambda_{m}\\ =F\Phi_{f}\left(\lambda_{m},\lambda_{x}\right)\Delta \lambda_{m}, \end{array}$$ (A9)

*A*_B_ (m^2^) is the cross section area of the intersection between the cylindrical volume illuminated by the incident laser beam and the source plane (see Fig. 4). The factor 4*π* is the total steridians into which the fluorescence is emitted and *f* is a angle-dependent function which describes possible non-isotropic emission. The solution index of refraction, *n*_S_, corrects for distortion due to refraction at the cuvette boundary. The factor *F* (1/m^2^sr) as defined in Eq. (9A), converts the total fluorescence radiant flux (W/nm) into fluorescence spectral radiance [W/(nm m^2^ sr)]. The conversion permits us to compare the fluorescence spectral radiance to standard sources whose properties are always defined in terms of spectral radiance. Using Eq. (A4), Eq. (A9) becomes

$$\begin{array}{c} L_{f}\left(\lambda_{m},\lambda_{x}\right)\Delta \lambda_{m}\\ =2.3FNly\left(\lambda_{m},\lambda_{x}\right)\Delta \lambda_{m}\varepsilon \left(\lambda_{x}\right)\Phi_{i}\left(\lambda_{x}\right)\Delta \lambda_{x}. \end{array}$$ (A10)

### 7.4 Measurement of the Fluorescent Radiance

The responsivity of the detection system is calibrated by a source of known spectral radiance *L*′ (*λ*_0_) [W/(m^2^ nm sr)]. This source can be either an integrating sphere with known spectral radiance at its exit port or a diffuse reflector, of known reflectance factor, illuminated by a source of known spectral irradiance. The emitting surface of the standard source coincides with the source plane in Fig. 4. Using Eq. (A8), the signal *S*′ (*λ*_0_) resulting from the source of known spectral radiance is

$$S^{\prime }\left(\lambda_{0}\right)=L^{\prime }\left(\lambda_{0}\right)\Gamma R\left(\lambda_{0}\right)\Delta \lambda_{m}.$$ (A11)

Likewise, the signal *S*_f_(*λ*_0_) resulting from the fluorescent source is

$$S_{f}\left(\lambda_{0}\right)=L_{f}\left(\lambda_{0}\right)\Gamma R\left(\lambda_{0}\right)\Delta \lambda_{m}.$$ (A12)

Taking the ratio of Eqs. (A11) and (A12) and using the expression for the fluorescent spectral radiance from Eq. (A10) yields

$$\begin{array}{c} L_{f}\left(\lambda_{0}\right)=\frac{S_{f}\left(\lambda_{0}\right)}{S^{\prime }\left(\lambda_{0}\right)}L^{\prime }\left(\lambda_{0}\right)\\ =2.3FNly\left(\lambda_{m},\lambda_{x}\right)\varepsilon \left(\lambda_{x}\right)\Phi_{i}\left(\lambda_{x}\right)\Delta \lambda_{x}. \end{array}$$ (A13)

Equation (A13) expresses the measured fluorescent spectral radiance *L*_f_ at a given wavelength setting of the detection system *λ*_0_ both in terms of measured or known quantities (*S*_f_, *S*′, and *L*′) and in terms of properties of the instrument (*F*, *l*, Φ_i_(*λ*_x_), and Δ*λ*_x_) and the sample (*N*, *y*, and *ε*).

The spectral quantum yield *y*(*λ*_m_, *λ*_x_) is conveniently separated into a quantum yield *ϕ* and a relative spectral emission function *s*(*λ*_m_, *λ*_x_) (1/nm), where

$$\int S\left(\lambda_{m},\lambda_{x}\right)d\lambda_{m}=1.$$ (A14)

The total fluorescent radiance *L*_f_ [W/(m2 sr)] is given by

$$L_{f}=\int L_{f}\left(\lambda_{0}\right)d\lambda_{0}.$$ (A15)

Substituting the right-hand expression in Eq. (A13) for *L*_f_(*λ*_0_) in Eq. (A15) and using Eq. (A14) yields the measurement equation relating the total fluorescent radiance to the appropriate instrument and sample properties, namely

$$L_{f}=2.3FNl\phi \varepsilon \left(\lambda_{x}\right)\Phi_{i}\left(\lambda_{x}\right)\Delta \lambda_{x}.$$ (A16)

In terms of measured and known quantities, the total fluorescent radiance, again using Eqs. (A15) and (A13), is

$$L_{f}=\int \frac{S_{f}\left(\lambda_{0}\right)}{S^{\prime }\left(\lambda_{0}\right)}L^{\prime }\left(\lambda_{0}\right)d\lambda_{0}.$$ (A17)

Equations (A16) and (A17) constitute the measurement model of fluorecence radiance. The measured fluorescence radiance [Eq. (A17)] is equated to the fluorescence radiance which results from known solution and measurement properties.
